# Supplementary material for: COZOID: contact zone identifier for visual analysis of protein-protein interactions
Source: BMC Bioinformatics. 2018 Apr 6;19:125. doi: 10.1186/s12859-018-2113-6 (PMC5889581; doi:10.1186/s12859-018-2113-6)
Supplement: Supplementary file 2 — Software build. Executable binary file of the software tool. (ZIP 118,784 kb) [file 12859_2018_2113_MOESM2_ESM.zip › cozoid/java/docs/beansbinding-1.2.1-doc.zip!/org/jdesktop/el/class-use/ELResolver.html]

Uses of Class org.jdesktop.el.ELResolver (Beans Binding)


---


|  |  |  |
| --- | --- | --- |
| **Overview** \| **Package** \| **Class** \| **Use** \| **Tree** \| **Deprecated** \| **Index** \| **Help** | |  |
| PREV   NEXT | **FRAMES**    **NO FRAMES**     **All Classes** |  |


---


## **Uses of Class org.jdesktop.el.ELResolver**

| Packages that use ELResolver | |
| --- | --- |
| **org.jdesktop.el** | Customized version of EL for Beans Binding; not for general use. |
| **org.jdesktop.el.impl.lang** | Customized version of EL for Beans Binding; not for general use. |

Uses of ELResolver in org.jdesktop.el

| Subclasses of ELResolver in org.jdesktop.el | |
| --- | --- |
| `class` | `ArrayELResolver`             Defines property resolution behavior on arrays. |
| `class` | `BeanELResolver`             Defines property resolution behavior on objects using the JavaBeans component architecture. |
| `class` | `CompositeELResolver`             Maintains an ordered composite list of child `ELResolver`s. |
| `class` | `ListELResolver`             Defines property resolution behavior on instances of `List`. |
| `class` | `MapELResolver`             Defines property resolution behavior on instances of `Map`. |
| `class` | `ResourceBundleELResolver`             Defines property resolution behavior on instances of `ResourceBundle`. |

| Methods in org.jdesktop.el that return ELResolver | |
| --- | --- |
| `abstract  ELResolver` | `ELContext.getELResolver()`             Retrieves the `ELResolver` associated with this context. |

| Methods in org.jdesktop.el with parameters of type ELResolver | |
| --- | --- |
| `void` | `CompositeELResolver.add(ELResolver elResolver)`             Adds the given resolver to the list of component resolvers. |

Uses of ELResolver in org.jdesktop.el.impl.lang

| Methods in org.jdesktop.el.impl.lang that return ELResolver | |
| --- | --- |
| `ELResolver` | `EvaluationContext.getELResolver()` |

---


|  |  |  |
| --- | --- | --- |
| **Overview** \| **Package** \| **Class** \| **Use** \| **Tree** \| **Deprecated** \| **Index** \| **Help** | |  |
| PREV   NEXT | **FRAMES**    **NO FRAMES**     **All Classes** |  |


---
